# Malate matters: disrupting bacterial-type phosphoenolpyruvate carboxylase (BTPC) rewires tomato fruit development

**DOI:** 10.1093/plphys/kiag026

**Published:** 2026-01-28

**Authors:** Felix J Martínez Rivas, Milena A Smith, Zahra Zangishei, Saleh Alseekh, Björn Usadel, William C Plaxton, Alisdair R Fernie

**Affiliations:** Max-Planck-Institute of Molecular Plant Physiology, Am Muehlenberg 1, Potsdam-Golm 14476, Germany; Department of Biology, Queen's University, Kingston, Ontario K7L 3N6, Canada; Faculty of Mathematics and Natural Sciences, Institute of Biological Data Science, CEPLAS, Heinrich Heine University Düsseldorf, Düsseldorf, Germany; Max-Planck-Institute of Molecular Plant Physiology, Am Muehlenberg 1, Potsdam-Golm 14476, Germany; Center for Plant Systems Biology and Biotechnology, Plovdiv 4000, Bulgaria; Faculty of Mathematics and Natural Sciences, Institute of Biological Data Science, CEPLAS, Heinrich Heine University Düsseldorf, Düsseldorf, Germany; Institute of Bio- and Geosciences IBG-4, Bioinformatics, BioSC, CEPLAS, Forschungszentrum Jülich, Jülich, Germany; Department of Biology, Queen's University, Kingston, Ontario K7L 3N6, Canada; Max-Planck-Institute of Molecular Plant Physiology, Am Muehlenberg 1, Potsdam-Golm 14476, Germany; Center for Plant Systems Biology and Biotechnology, Plovdiv 4000, Bulgaria

## Abstract

Plant phospho*enol*pyruvate carboxylases (PEPCs) are ubiquitously expressed as cytosolic Class-1 PEPC homotetramers composed of 107 kDa plant-type PEPC (PTPC) subunits that are highly sensitive to allosteric inhibition by malate. Class-2 PEPC heterooctameric complexes that are desensitized to malate inhibition also exist in certain sink tissues due to the interaction of a Class-1 PEPC with unrelated 118 kDa bacterial-type PEPC (BTPC) polypeptides. Class-2 PEPCs dynamically associate with the mitochondrial outer envelope and have been hypothesized to support sustained anaplerotic flux and respiratory CO₂ refixation in malate-rich sink tissues, including immature tomato fruit. The current study generated *CRISPR-Cas9*-edited tomato lines with targeted disruption of the *BTPC* gene and investigated the impact on fruit development, metabolism, and transcriptional regulation. Immunoblotting and co-immunoprecipitation confirmed the absence of BTPC polypeptides and Class-2 PEPC complexes in the edited lines. Fruits from the edited plants were 25% smaller and 40% lighter and required up to 10 additional days to complete ripening compared to the WT. Metabolomic analysis across ripening stages revealed substantial reductions in malate and citrate, with elevated sugars and amino acids, indicating reprogrammed carbon flux. RNA-seq data showed downregulation of genes for cell wall remodeling, sugar transport, and ethylene-responsive transcription factors. These results provide direct evidence that BTPC is essential for organic acid balance, sugar metabolism, and ripening regulation in tomato. Its absence perturbs metabolic homeostasis and developmental progression, positioning BTPC as a strategic target for enhancing fruit quality traits through genetic engineering.

## Teaser

Loss of bacterial type phosphoenolpyruvate carboxylase reprograms fruit metabolism, altering sugar/acid balance, and delaying ripening through disrupted carbon flux and cell wall remodeling.

## Introduction

Tomato (*Solanum lycopersicum*) is a model organism for studying fleshy fruit development and ripening (Klee and Giovannoni 2011). The ripening process involves coordinated developmental, metabolic, and hormonal changes that transform immature, photosynthetically active green fruits into soft, sweet, and flavorful red fruits. Early in development, green tomato fruits are partially autotrophic, accumulating photosynthates as organic acids and starch, while during ripening, there is a shift to a heterotopic metabolism, which is accompanied by rapid compositional changes (Lytovchenko et al. 2011; Batista-Silva et al. 2018). During tomato ripening, sugars accumulate via import from source tissues, breakdown of starch and gluconeogenesis, while organic acids that are abundant in green stages decline due to their metabolization to obtain the energy required to support the multiple metabolic changes that occur during climacteric ripening (Carrari 2006; Carrari et al. 2006; Fait et al. 2008; Osorio et al. 2013; Beauvoit et al. 2018; Martínez-Rivas and Fernie 2024). This coordinated reprogramming of central metabolism results in increased sweetness and decreased tartness of ripe tomatoes. Indeed, the balance between organic acids and sugars is critical for defining fruit taste, with the ratio of sugars/acids being the main determinant of consumer preference and market value (Kader 2008). Interestingly, most efforts aimed at breeding sweeter tomatoes came with an unwanted yield penalty, with the trade-off between sweetness and yield only recently being broken and even more recently mechanistically understood (Zhang et al. 2024; Fernie and Martinez-Rivas 2025). The afore-mentioned shifts in metabolite levels are integrated with the rest of the ripening process on time, being regulated by ethylene and transcription factors, and are crucial to support the fruit's energetic demands (Lanahan et al. 1994; Giovannoni et al. 1995; Barry and Giovannoni 2006). Comparative studies demonstrate that other fleshy fruits follow a similar pattern of rising sugars and declining organic acids during the ripening process—even in non-climacteric fruits such as pepper (Klie et al. 2014; Batista-Silva et al. 2018; Martínez-Rivas and Fernie 2024). This rebalancing of carbon metabolism is not only important for fruit quality but also reflects the physiological adjustments made by the fruit in the transition from a growing, organic acid-rich organ to a mature sugar-rich organ.

Beyond describing the fluctuation of metabolites' levels, functional studies have underscored the importance of organic acid metabolism for fruit development and ripening in tomato. For example, the work by Centeno et al (2011). demonstrated that modifying malate levels via the specific reduction of malate dehydrogenase or fumarase in the fruit had profound effects on fruit metabolism, altering starch and sugar accumulation and impacting postharvest disease resistance. Similarly, tomato plants exhibiting decreased cytosolic phospho*enol*pyruvate (PEP) carboxykinase (PEPCK) and plastidic NADP ^+^ -dependent malic enzyme (ME) displayed strongly altered fruit metabolism, with both transgenic lines characterized by decreased starch metabolism, but only cytosolic PEPCK lines exhibiting an altered respiration rate (Osorio et al. 2013). Moreover, changes in 2-oxoglutarate dehydrogenase (OGDH) and mitochondrial isocitrate dehydrogenase showed a disbalance in organic acid and sugar patterns, as well as amino acid levels (Araújo et al. 2010, 2011, 2012a, b; Sulpice et al. 2010), indicating that changes in organic acid metabolism and tricarboxylic acid (TCA) cycle flux trigger metabolic reprogramming during development and ripening. A relatively commonly observed phenotype in the mutant lines displaying altered balance between organic acids and sugars was the alteration of fruit size (although this was not observed in the PEPCK and plastidic NADP ^+^ -dependent ME lines). These phenotypes thus largely validate the postulated role of organic acids in fruit growth, with cell growth and expansion relying on osmotic potential for cell enlargement, and this is largely determined by sugars and acids (Liu et al. 2007). The metabolic network of tomato fruit is highly responsive to changes in organic acid levels, which renders the understanding of the control of the balance between acids and sugars while fruit ripening highly important. Recent metabolomics-driven analyses reinforce this concept, revealing conserved changes in central metabolites such as sugars, acids, and amino acids across developmental stages and providing a systems-level view of ripening-associated metabolic regulation (Klie et al. 2014; Martínez-Rivas and Fernie 2024).

A crucial enzyme that participates in the control of central carbon flux and organic acid metabolism is PEP carboxylase (PEPC, EC 4.1.1.31), which catalyzes the irreversible β-carboxylation of PEP in the presence of HCO_3_^−^ to yield oxaloacetate (OAA) and inorganic phosphate. PEPC is present in all plant tissues and is known for its dual role in photosynthetic and non-photosynthetic metabolism. In C4 and CAM photosynthetic tissues, PEPC plays the crucial role of fixing atmospheric CO_2_, providing OAA, or subsequently malate or aspartate as a CO_2_ shuttle in carbon assimilation (Chollet et al. 1996; O’Leary et al. 2011b). However, PEPC also plays an important anaplerotic role in all tissues, by replenishing TCA intermediates consumed in anabolic processes (O’Leary et al. 2011b). Thus, in fruits, seeds, and roots, PEPC-derived OAA and/or malate are critical for maintaining energy metabolism and redox balance.

Plant PEPC is encoded by a small gene family comprising plant-type PEPC (PTPC) and bacterial-type PEPC (BTPC). Most plant PEPC isoforms exist as Class-1 PEPC homotetramers composed of 107 kDa PTPC polypeptides and are subject to tight regulation by allosteric effectors (O’Leary et al. 2011a). Class-1 PEPCs are activated by glucose-6-phosphate and potently inhibited by L-malate, aspartate, and glutamate (Echevarria and Vidal 2003). Their activity is also subject to reciprocal control by phosphorylation for activation and monoubiquitation for inhibition at highly conserved serine and lysine residues, respectively (Echevarria and Vidal 2003; Uhrig et al. 2008b; O’Leary et al. 2011a; Ruiz-Ballesta et al. 2016). On the other hand, immunologically unrelated BTPC isoforms, which were initially discovered through biochemical and/or genomic analyses of green microalgae (*Selenastrum minutum* and *Chlamydomonas reinhardtii*) (Rivoal et al. 2001; Mamedov et al. 2005) and vascular plants (Arabidopsis, castor, rice, and soybean) (Blonde and Plaxton 2003; Sánchez and Cejudo 2003; Sullivan et al. 2004), form a monophyletic PEPC clade (O’Leary et al. 2011b). BTPC polypeptides share a higher sequence similarity with prokaryotic PEPCs than with PTPCs (Sánchez and Cejudo 2003; Sullivan et al. 2004; Mamedov et al. 2005), with at least one *BTPC* gene occurring in all vascular plant genomes sequenced to date, alongside multiple *PTPC* genes (O’Leary et al. 2011b; Ting et al. 2017). Plant *BTPC* genes encode approximately 118 kDa polypeptides that contain a unique intrinsically disordered domain that mediates their tight association with co-expressed PTPC polypeptides. This interaction results in the formation of unusual Class-2 PEPC hetero-octameric complexes that were first purified and biochemically characterized from *Chlamydomonas, Selenastrum*, and the endosperm of developing castor oil seeds (Rivoal et al. 2001; Blonde and Plaxton 2003; Mamedov et al. 2005; Tripodi et al. 2005; Gennidakis et al. 2007; Uhrig et al. 2008a). BTPCs have diverged from PTPCs in sequence and regulation and appear to mainly be expressed in malate-accumulating sink tissues (Ting et al. 2017). BTPC functions as a catalytic and regulatory subunit of Class-2 PEPCs, by not only contributing to catalytic PEPC activity but also greatly dampening the inhibitory effect of allosteric effectors such as malate on associated PTPC subunits within the complex (O’Leary et al. 2009, 2011a). This property is of special relevance in sink tissues, such as developing tomato fruit, where malate accumulates to very high levels. Furthermore, in contrast to the strictly cytosolic Class-1 PEPC, Class-2 PEPC has been localized to the outer mitochondrial envelope *in vivo*, an interaction mediated by its BTPC subunits (Park et al. 2012). In developing castor seeds and tomato fruit, Class-2 PEPC was hypothesized to refix respired CO_2_ while supporting the anaplerotic replenishment of TCA cycle intermediates withdrawn for anabolism without being inhibited by the very high malate levels present in these tissues (O’Leary et al. 2011a, b; Park et al. 2012; Ting et al. 2017).

In light of the above description, ripening tomato fruit—as a malate-rich and non-photosynthetic sink tissue—is a good model in which to study BTPC. Previous studies determined that BTPC, which is encoded as a single gene in tomato, reaches its maximum expression in mature green fruits (Ting et al. 2017). Immunoblot and co-immunoprecipitation (co-IP) studies confirmed that tomato fruit contains a 118 kDa BTPC that physically interacts with 107 kDa PTPC subunits, forming a Class-2 PEPC complex analogous to that occurring in developing castor oil seeds (Ting et al. 2017). We previously postulated that the presence of a BTPC-containing Class-2 PEPC complex in immature tomato fruits represents an adaptive mechanism that sustains PEPC activity despite the high malate levels occurring in this tissue. Despite this hypothesis, the *in vivo* role of BTPC in fleshy fruits remains to be explored.

The current study functionally characterizes the role of BTPC in tomato fruits by using CRISPR-Cas tomato lines in which the *BTPC* gene was edited. We used a wide range of phenotypical, metabolomic, and transcriptomic techniques to analyse the edited lines in order to determine how the loss of the BTPC enzyme affects tomato fruit development and ripening. Our results are discussed with regard to the above-mentioned hypothesis regarding the role of Class-2 PEPC. In summary, by elucidating the function of BTPC, we gain further insight into the control of organic acid metabolism, which is of broad relevance in plant metabolic biology, providing a deeper perspective on how plants modulate central carbon metabolism under different conditions through specialized enzyme isoforms.

## Results

### Generation of CRISPR/Cas9 *slbtpc* mutants

Ting and co-workers (2017) reported that the tomato genome contains a single BTPC gene (*SlBTPC*, Solyc04g00697.2), predicted to encode a 117.9 kDa polypeptide. Sequence alignment revealed that all residues important for catalytic PEPC activity are conserved in the deduced SlBTPC polypeptide, as well as: (i) the intrinsically disordered domain and C-terminal (R/K)NTG tetrapeptide characteristic of BTPCs, and (ii) regulatory (inhibitory) Ser425 & Ser451 phosphosites that have been identified and studied in castor BTPC (RcPPC4) (Fig. S1) (O’Leary et al. 2011a; Dalziel et al. 2012; Ying et al. 2017). *SlBTPC* is maximally expressed during the immature stages of tomato fruit development, peaking at the mature green stage and decreasing at breaker and red stages (Fig. S2, Ting et al. 2017; Shinozaki et al. 2018). In order to investigate the contribution of *SlBTPC* to tomato fruit development, we generated CRISPR/Cas9 targeted mutations in this gene. After transformation, more than 30 transgenic shoots were obtained, and three of them were selected for further studies after screening. We selected lines that lacked the Cas9 (to prevent further mutations) and that contained the homozygous mutation on the *SlBTPC* sequence. Line 7 (L7) and L8 displayed a single bp insertion, and L13 a two bp deletion. Both these modifications induced premature stop codons at positions 54 and 80, respectively, leading to a non-functional SlBTPC polypeptide (Fig. S3). These three lines were used for further studies.

### Validation of BTPC “knockout’ in *slbtpc* CRISPR-Cas9 mutant fruit

**Figure 1:**
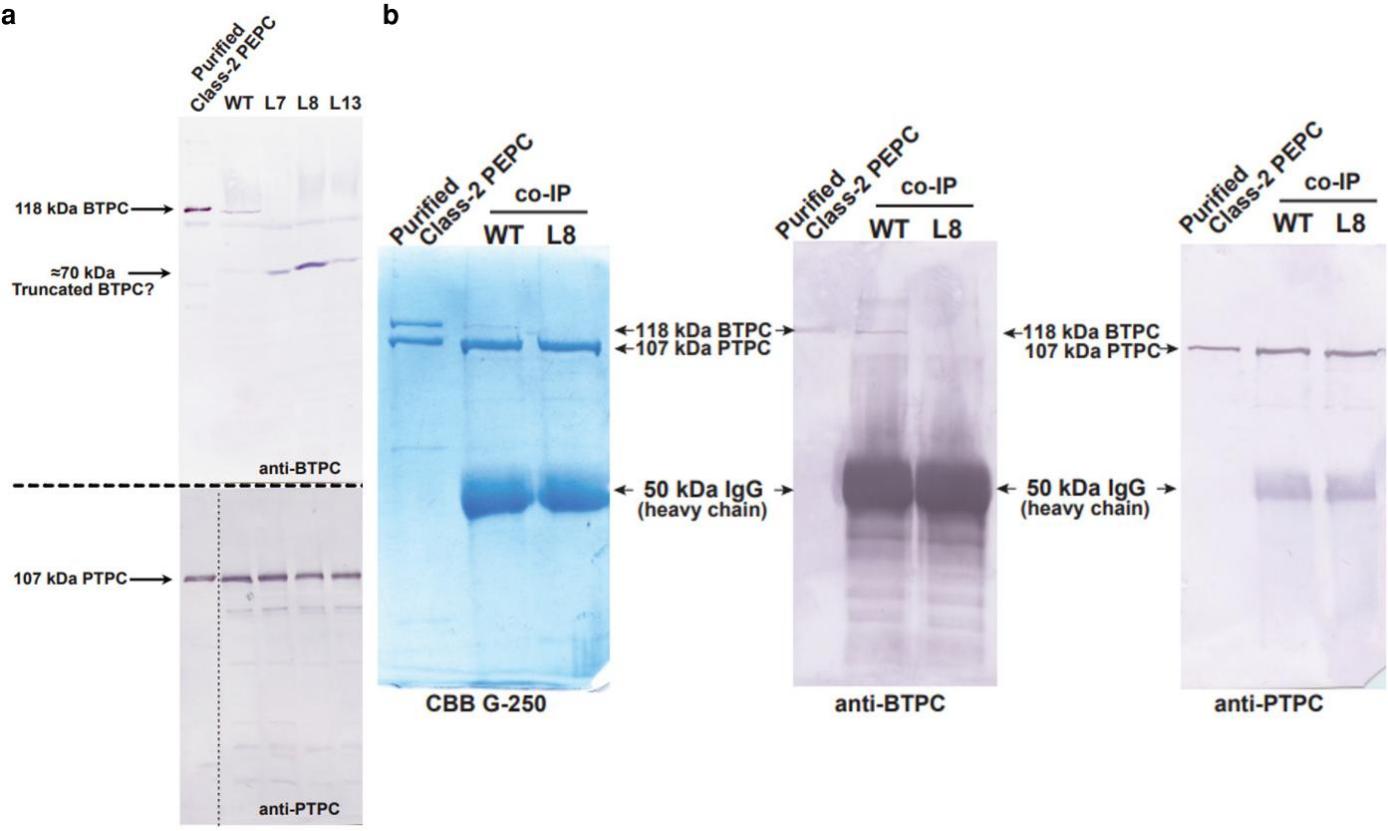
Loss of bacterial-type phosphoenolpyruvate carboxylase disrupts class-2 PEPC complex formation in tomato fruits. **(a)** Immunoblot analysis of PTPC and BTPC polypeptides in WT and transgenic tomato fruit lines. Lane 1 contains purified, recombinant chimeric Class-2 PEPC (100 ng) consisting of a 1:1 ratio of 118 kDa RcPPC4 (castor BTPC) and 107 kDa AtPPC3 (an Arabidopsis PTPC isozyme) polypeptides (O’Leary et al. 2009), along with clarified extracts from immature green tomato fruit, which were subjected to SDS-PAGE and immunoblotting using anti-PTPC and anti-BTPC (15 and 50 µg of protein loaded per lane, respectively). Antigenic polypeptides were visualized using a phosphatase-conjugated secondary antibody and chromogenic staining. **(b)** Co-immunoprecipitation of BTPC and PTPC polypeptides from WT and L8 immature tomato fruit. Co-IP was performed using purified anti-PTPC IgG as described in the Materials and Methods. Co-IP eluates were subjected to SDS-PAGE followed by Coomassie Blue G-250 staining or immunoblotting with anti-PTPC and anti-BTPC as indicated. Lane 1 contains purified recombinant chimeric Class-2 PEPC (100 ng) as a positive control (O’Leary et al. 2009); all other lanes contain 15 µL each of the respective co-IP eluates. WT: Wild type plants, L8 Line 8.

Loss of full-length (118 kDa) SlBTPC polypeptides in the three mutant lines was verified by immunoblotting immature green fruit extracts with anti-(castor BTPC). Immunoreactive 118 kDa polypeptides that co-migrated with castor BTPC were observed for the wild type (WT), but not *CRISPR-cas9* edited lines (Fig. 1a). By contrast, an approximate 70 kDa immunoreactive polypeptide was evident on the anti-BTPC immunoblots of the CRISPR-Cas9 mutant, but not WT, fruit extracts (Fig. 1a). Furthermore, there were no obvious differences in the abundant levels of 107 kDa anti-(castor PTPC) immunoreactive polypeptides in the immunoblots of the edited, relative to WT fruit extracts (Fig. 1a). Similarly, PEPC activity assays revealed that there were also no significant differences in the PEPC activity of the corresponding immature fruit extracts (whether normalized in terms of mg protein or gDW) (Fig. S4). This is likely because PTPC expression (& thus Class-1 PEPC formation) is much greater than BTPC expression (and thus Class 2 PEPC formation) in the WT fruit.

Co-IP using anti-PTPC was also performed on extracts prepared from immature fruit of WT versus L8 *slbtpc*-edited plants followed by SDS-PAGE and anti-BTPC versus anti-PTPC immunoblotting of the co-IP eluates. Activity assays confirmed that all PEPC activity was effectively immunoprecipitated from the respective extracts. The co-IP results corroborate those of Ting et al. (2017) that BTPC associates with PTPC to form a Class 2 PEPC complex in WT fruit since 118 kDa BTPC polypeptides co-IP’d with 107 kDa PTPC polypeptides (Fig. 1b). By contrast, only immunoreactive 107 kDa PTPC polypeptides were evident following anti-PTPC versus anti-BTPC immunoblotting of co-IP eluates prepared from the L8 edited fruit.

### *SlBTPC* mutant lines produced smaller fruits with delayed ripening time

**Figure 2:**
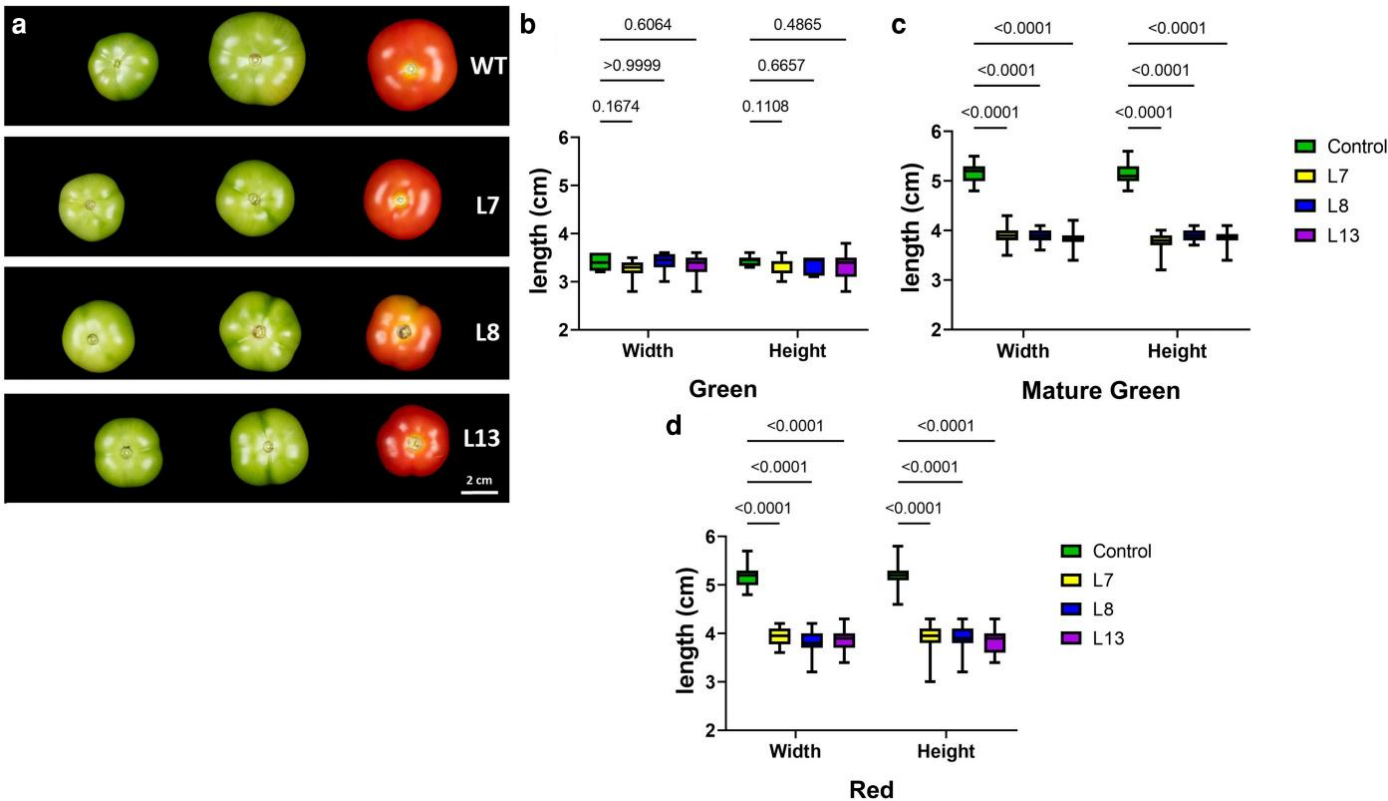
Differences in the size of edited fruits were observed along development and ripening. **(a)** General overview of the fruits at different ripening stages. Width and height of WT and *slbtpc*-edited tomato fruits at immature green **(b)**, mature green **(c)**, and Red stages **(d)**. Data are shown as Box-and-Whisker plots (line = median, box = 25–75th percentile, whiskers = min–max). Statistical significance was determined by one-way ANOVA followed by Dunnett's *post hoc* test comparing each mutant to WT. *n* > 25 replicates per genotype. WT: Wild type, L7: Line 7, L8: Line 8, L13: Line 13.

**Figure 3:**
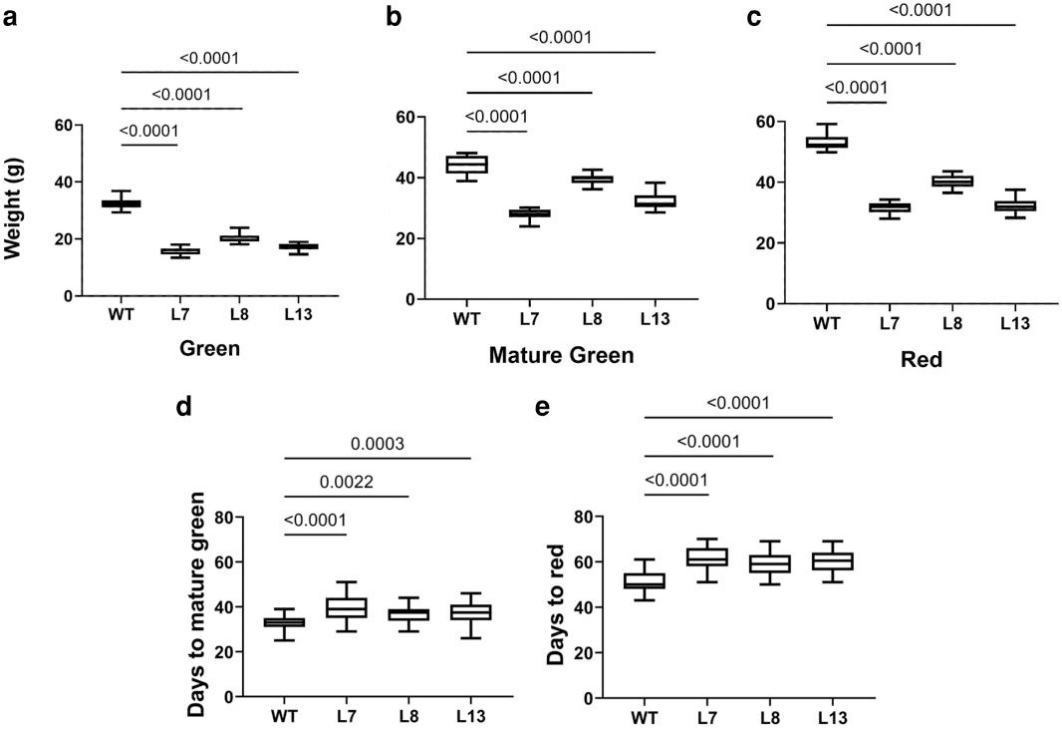
Phenotypical differences between WT and *slbtpc-*edited lines. a, b, and c depict fruit fresh weight at immature green, mature green, and red stages, respectively. At least 20 fruits per plant were harvested at different developmental stages and weighed per line. d and e number of days to reach the mature green and full red stage. Data are shown as Box-and-Whisker plots (line = median, box = 25–75th percentile, whiskers = min–max). Statistical significance was determined by one-way ANOVA followed by Dunnett's *post hoc* test comparing each mutant to WT. *n* > 20 flowers were tagged per line on anthesis day per genotype.

CRISPR/Cas9 edited lines presented no obvious vegetative differences in either growth rate or vegetative morphology (Fig. S5). Moreover, there were no differences in flowering time between the edited lines and the WT. Nonetheless, once the plants started to produce fruits, we noticed that the *slbtpc*-edited lines produced smaller fruits, which were accompanied by a clear delay in the ripening phase. Fruits from the edited lines displayed a similar size at the early stages of development to those of the WT (Fig. 2a and b). However, their development stalled, as their increase in size was considerably slower when compared to WT. Once the fruits reached their maximum size, namely at the mature green stage, fruits from the edited lines were up to 25% smaller than those of the WT (Fig. 2c), a difference of size that was also maintained in the red stage (Fig. 2d). Fruits of the edited lines were not only smaller, but they also weighed less than WT fruit (Fig. 3a). At the mature green stage, fruits of the *slbtpc*-edited lines were approximately 15 g lighter than those of the WT (Fig. 3b). This difference was increased at the red stage, where fruits of the edited lines weighed around 20 g less than those of the WT (Fig. 3c). These results suggest that the knock-out of SlBTPC is affecting the rate of assimilation of and/or import into the fruits, and consequently, the edited lines produce smaller and lighter fruits.

Furthermore, the number of days to reach the mature green stage and thereby complete ripening was recorded. Consistent with the production of smaller fruits, fruits from the gene-edited lines took longer to develop and ripen. As seen in Fig. 3d, fruits from the gene-edited lines took an average of 6 days longer to reach the mature green stage. This difference was higher in the time frame needed to reach the ripe stage. Fruits from the *slbtpc-*edited lines took on average 10 days longer to reach this stage (Fig. 3e), which clearly indicates a hindrance on the development and ripening.

### Mutation of SlBTPC alter central metabolism in tomato fruits

**Figure 4:**
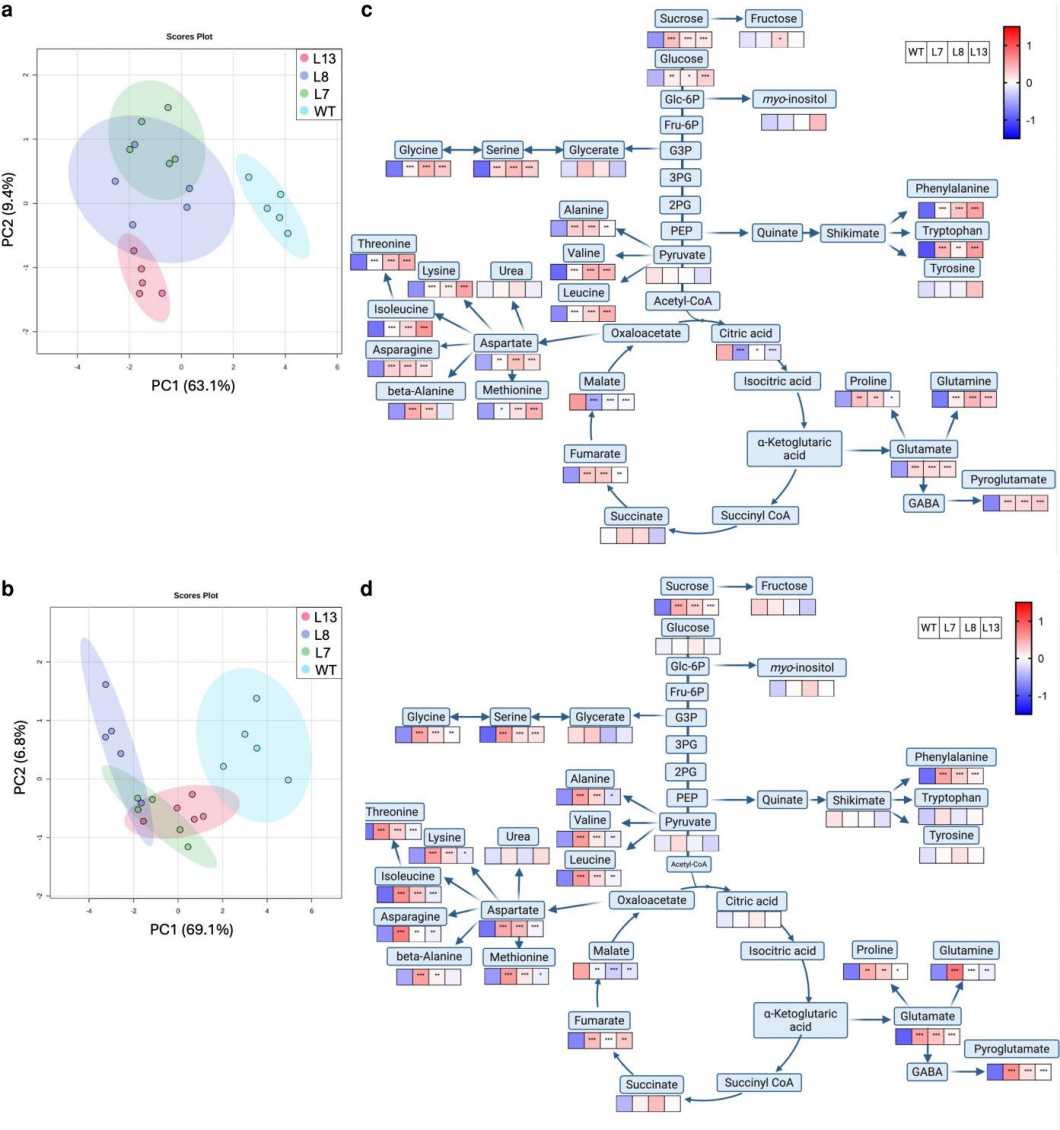
Overview of metabolic results at mature green and red stages. a and b depict the PCA analysis from the mature green and red stages, respectively. c and d show metabolite levels at mature green and red stages, respectively. Pools of three fruits per plant and five plants per line were used. Metabolite levels were log-transformed and pareto scaled for normalization. The scale ranges from low relative abundance (blue) to high relative abundance (red). Statistical significance was determined by one-way ANOVA followed by Dunnett's *post hoc* test comparing each mutant to WT. Asterisks indicate statistically significant differences: *P < 0.05; **P < 0.01, ***P < 0.001. ****P < 0.0001.

Given the key role of PEPC in central metabolism, we next performed metabolic profiling using GC-MS on samples harvested from WT and the three edited lines at the three different ripening stages already mentioned. A total of 39 primary metabolites were identified, including sugars, organic acids, and amino acids, which were differentially accumulated in the *slbtpc* edited fruits compared to WT (Table S1). A principal component (PCA) analysis revealed that in the immature green stage, there was no clear separation between fruits from the gene-edited lines and the WT (Fig. S6a). However, in mature green and red stages, fruits from the gene-edited lines and the WT were clearly distinguishable, with biological replicates clustering within each genotype (Fig. 4a and b).

At green stages, even though there is no clear separation between genotypes according to the PCA, some metabolites present statistically different accumulation. For example, BTPC edited fruits accumulate higher levels of sucrose and fumarate, compared to WT fruits (Fig. S6b). Conversely, citrate and malate presented a clear reduction, suggesting an early disruption in TCA cycle flux. An overview of the changes in the different amino acids revealed a decrease in proteinogenic amino acids such as isoleucine, threonine, and leucine. While no changes were observed in pyruvate, the level of its derivatives -alanine, valine, and leucine—clearly increased, suggesting a shift from that of the classical TCA cycle to that of branched chain amino acid synthesis (Araújo et al. 2010; Sweetlove et al. 2010).

The metabolic differences became more pronounced at the mature green stage, a critical point during fruit ripening with a notable depletion in organic acids, especially malate and citrate in fruits from the gene-edited lines (Fig. 4c). By contrast, sugars such as sucrose, glucose, and fructose were present in higher levels in the fruits of the *slbtpc* edited lines, which suggests an altered carbon flux, favoring sugar accumulation over TCA cycle intermediates. Amino acid metabolism was dramatically altered, with levels of all quantified amino acids increasing in the edited lines. For example, we observed several fold increases in valine, alanine, and leucine, in the absence of changes in the level of pyruvate, suggesting a re-routing of carbon flux towards amino acid biosynthesis at the cost of energy metabolism.

A sustained alteration in central metabolism during fruit ripening is maintained, according to the metabolic changes observed at the red stages (Fig. 4d). Consistent with in earlier stages, ripe fruit from the *slbtpc* edited lines displayed a clear reduction in citrate and malate, reinforcing the hypothesis that TCA cycle activity could be compromised. This suggests a redirection of carbon fluxes that could modulate mitochondrial respiration and disturb the overall energetic balance within the fruit tissues. Carbohydrate accumulation was also maintained, like in mature green fruit, sucrose, glucose and fructose exhibited significantly higher levels in fruits from the edited lines relative to WT. This trend was also followed by all amino acids determined which maintained their higher levels as occurred at the mature green stage.

### Transcriptomic alterations in BTPC edited fruits at mature green stage

**Figure 5:**
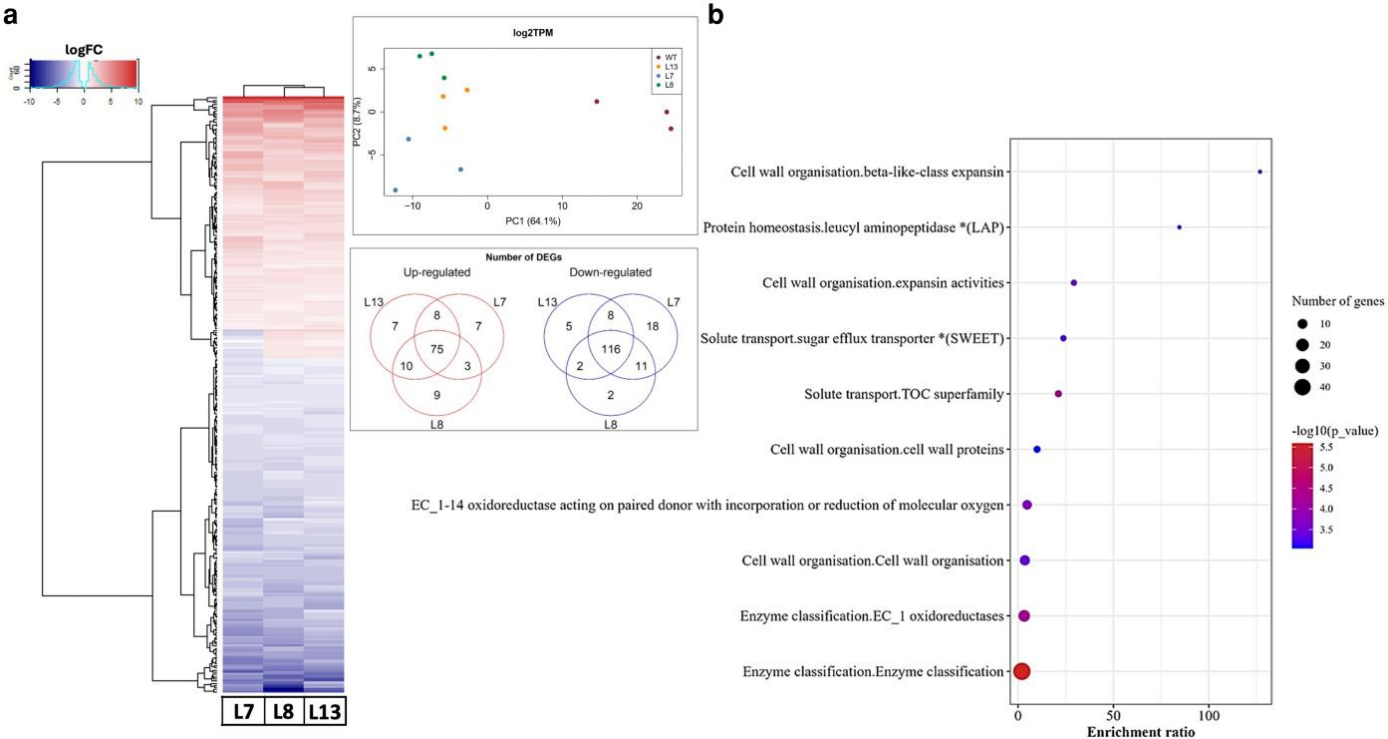
Overview of transcriptomics analysis at the mature green stage. **(a)** Right 20% of the differentially expressed genes (DEGs) are based on FDR with |LFC| ≥ 1 in at least one of the three lines. Left PCA analysis with the DEG genes. Venn diagram with common and unique up- and downregulated genes. **(b)** Top 10 Over-represented categories for the down-regulated group of the DEG.

**Figure 6:**
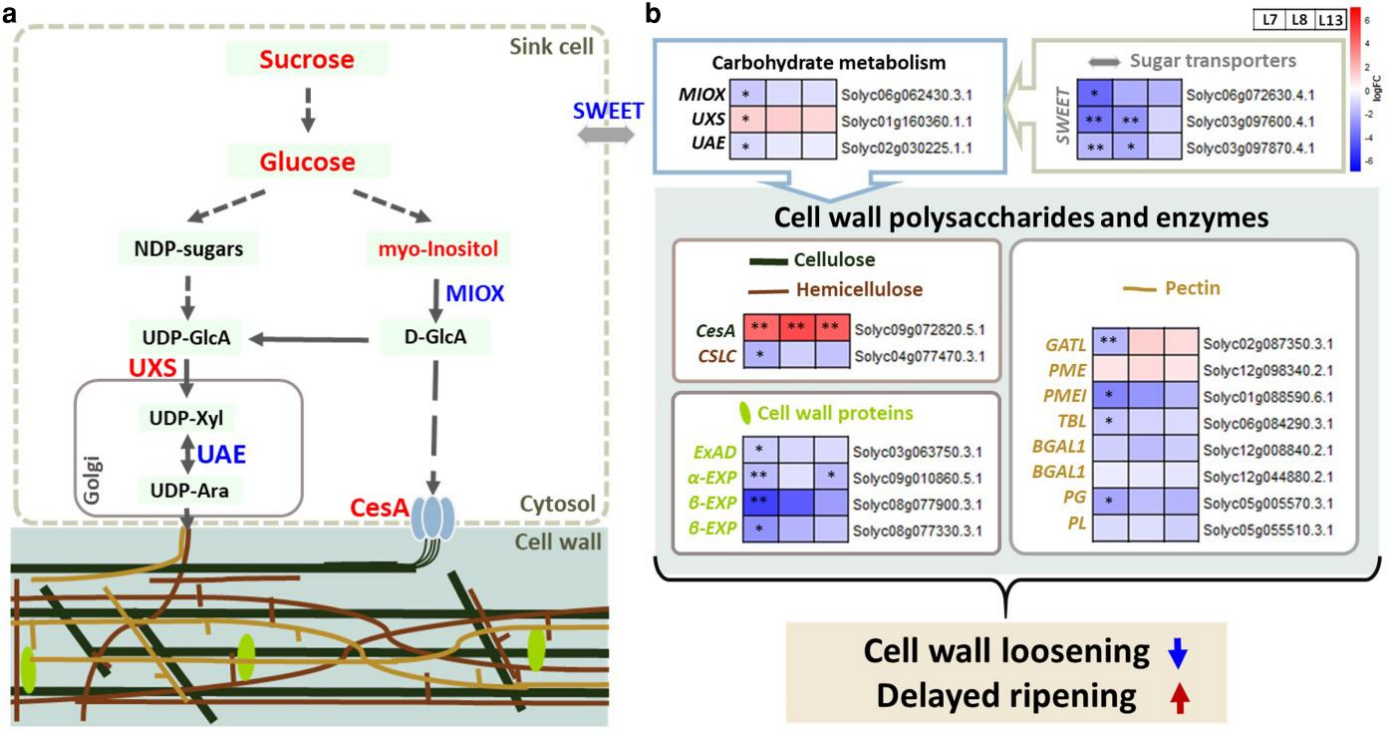
Reprogramming of sugar metabolism and cell wall remodeling pathways in *slbtpc* tomato fruits. **(a)** Simplified scheme of the sugar allocation toward the cell wall remodelling. Only differentially regulated genes are shown. Red and blue indicate up-regulated and down-regulated metabolite or enzyme, respectively. Solid line represent a single metabolic step, dashed lines mean multiple steps. **(b)** Workflow representing the possible correlation of the reprogramming of the genes related to sugar metabolism and cell wall organization with delayed ripening. ** and * indicate FDR < 0.01 and FDR < 0.05, respectively. sugar efflux transporter (SWEET), myo-inositol oxygenase (MIOX), UDP-D-glucuronic acid decarboxylase (UXS), UDP-Ara 4-epimerase (UAE), catalytic component (CesA) of cellulose synthase complex (CSC), Galacturonosyltransferase-Like (GATL), 1,4-beta-glucan synthase (CSLC), pectin methylesterase (PME), pectin methylesterase inhibitor (PMEI), rhamnogalacturonan-I O-acetyltransferase (TBL), beta-galactosidase (BGAL1), regulatory protein of polygalacturonase activity (PG), pectate lyase (PL), alpha-1,3-arabinofuranosyltransferase (ExAD), alpha-class expansin (α-EXP), beta-like-class expansin (β-EXP).

To gain further insight into the molecular mechanisms that underlie the altered metabolic and phenotypic changes observed in fruits of the *slbtpc*-edited lines, we performed RNAseq analysis on fruit tissues collected at the mature green stage from three independent edited lines and the WT. Differentially expressed genes (DEGs) based on a False Discovery Rate of 20% and with a |log_2_FC| > 1 in at least one of the lines allowed the detection of 274 genes. PCA indicated a differential transcriptional pattern for these genes between the edited lines and the WT (Fig. 5a). A detailed description of the biological functions for the genes was gained using the Mapman4 functional annotation (Schwacke et al. 2019) (Table S2). Amongst the top enriched classes for the down-regulated group of the DEGs (Fig. 5b), cell wall remodeling and efficient sugar allocation were noticeable, reflecting in a slower fruit development and ripening. Of the categories representing the downregulated genes, the family of expansins (EXPs), polygalacturonase (PG), pectate lyase (PL), pectin methylesterase inhibitor (PMEI), and beta galactosidase (BGAL1), were observed (Fig. 6). These genes are markedly downregulated in the edited fruits at mature green stage, when the cell wall is supposed to loosen its rigidity and allow the fruit to grow.

Moreover, among the strongly upregulated genes, we observed cellulose synthase (CesA), which suggests an increase in cellulose biosynthesis, reinforcing the idea of a rigid cell wall that impedes fruit growth. The imbalance between pectin degradation (suppressed) and cellulose biosynthesis (enhanced) likely contributes to the altered softening process in fruits of the edited lines. Moreover, other genes related to cell expansion promotion, such as those of the SAUR family, were also downregulated, suggesting a global transcriptomic reprogramming that impedes cell wall loosening and cell division (Fig. 6).

Genes pertaining to 2-oxoglutarate metabolism or glycolysis were also repressed. Glutamate dehydrogenase, threonine deaminase, and enolase were downregulated in fruits from the *slbtpc*-edited lines, which aligns with the observed lower malate and citrate levels (Fig. S7 and Table S2). Moreover, sugar efflux also appeared to be impaired. Three members of the SWEET transporters and some glycosyltransferases were also downregulated. These shifts might be responsible for the reduction of sugar mobilization, resulting in elevated intracellular glucose and fructose (Fig. 6). Among the downregulated genes, we also observed reduced expression related to volatile biosynthesis, such as methyl esterases, carboxylesterases, and phenylacetaldehyde reductase (Table S2).

Among the up-regulated DEGs, the marked induction of cellulose synthase (CesA) transcripts was particularly notable, suggesting enhanced cellulose biosynthetic activity. Consistent with the extensive downregulation of genes involved in cell wall organization, including expansin-like proteins, β-expansin family members, pectin methylesterases (PMEs) and their inhibitors (PMEIs), polygalacturonases (PGs), pectate lyases (PLs), and cell wall proteins, these changes point to an imbalance in cell wall remodeling capacity during fruit development. These transcriptional data point to a less dynamic cell wall metabolism, potentially contributing to reduced fruit expansion and delayed tissue softening.

Interestingly, on top of the list for strongly up-regulated genes is the gene encoding the exocyst complex components (SEC 3), one of the key subunits that functions in exocytosis as the central mechanism of cell morphogenesis (Fig. S8). Although the direct evidence is lacking, given the conservation of exocyst function in processes requiring targeted exocytosis such as primary and secondary cell wall deposition (Kulich et al. 2010; Hoffmann et al. 2021), SEC3 is very likely to be involved in directing vesicle traffic concerned with the polarized deposition of cellulose and pectins toward the cell wall integrity.

Furthermore, the next most strongly upregulated gene in fruits of the edited lines is that encoding the aromatic amino acid decarboxylase AADC1A (Fig. S8), which is associated with the flavor in tomato fruits. Converting phenylalanine to phenethylamine, AADC1A mediates the first step in the production of phenylalanine-derived volatiles (Tieman et al. 2006).

Genes related to amino acid metabolism, such as transporters and genes involved in protein degradation or proteostasis, such as E3 ubiquitin ligases and conjugating enzymes, were upregulated. This suggests both enhanced nitrogen flux towards amino acids or its redistribution, as well as an active proteolytic response in the fruit of the gene-edited lines. Taken together, the transcriptomic analysis of the *slbtpc*-edited fruits reveals a considerable reprogramming of the key ripening-associated pathways, which is tightly connected with the metabolic results. Repression of genes encoding TCA cycle enzymes and other genes that influence organic acid metabolism aligns with the lower levels of malate and citrate in the fruit of the gene-edited lines, while the suppression of the amino acid catabolic enzymes is consistent with the elevated amino acid levels in these lines. Most of the cell wall organization-related genes are downregulated, corresponding to the impaired cell wall loosening and reduced fruit size, while SWEET transporter downregulation could explain the higher sugar concentration (Zhang et al. 2024). Additionally, these data might support a model in which the targeted exocytosis contributes to the cell wall deposition in edited fruits. Altogether, these data underscore the central regulatory role of BTPC in fruit energy balance, carbon partitioning, and developmental timing, offering insights into metabolic control during tomato ripening.

## Discussion

BTPC isoforms have been identified in several green microalgae and vascular plant species (Rivoal et al. 2001; O’Leary et al. 2011b; Ting et al. 2017), but to date, the most extensive work has been performed in developing castor oil seeds. It has been reported that BTPC subunits tightly interact with typical PTPC polypeptides to form an unusual hetero-octameric Class-2 PEPC complex that is desensitized to allosteric inhibitors and that dynamically associates with the mitochondrial surface (O’Leary et al. 2009; Park et al. 2012). Class-2 PEPCs are thereby believed to enable rapid refixation of respired CO_2_ while sustaining anaplerotic flux of OAA into the TCA cycle, generating the energy and C-skeletons needed to support the biosynthetic demands of developing sink tissues that accumulate high levels of malate (O’Leary et al. 2011a; Ting et al. 2017). In C4 and CAM photosynthesis, the PTPC containing Class-1 PEPC homotetramer has an important role in fixing atmospheric CO_2_ that has been extensively reviewed elsewhere (Chollet et al. 1996; O’Leary et al. 2011a). By contrast, studies of PEPC's role in non-photosynthetic tissues, by knocking out its expression, have received far less attention to date. Indeed, this has been largely confined to the study of sorghum plants with RNAi targeted to *SbPPC3, a* root-specific PTPC isoform with the resultant edited plants being characterized by reduced plant size and seed production, delayed flowering, an accumulation of amino acids, and impaired phosphate acquisition (de la Osa et al. 2022; Pérez-López et al. 2026). A further interesting observation in these *sbppc3* edited plants was that they accumulated Asn and Gln as the plants shifted excess nitrogen into amides when the carbon supply from PEPC was limiting. Similar results were obtained in our study of *slbtpc* edited lines, where we observed that the mutant plants produced smaller fruits with delayed ripening and an increase in the levels of almost all measured amino acids. However, to our knowledge, the effects of downregulation of BTPC have yet to be characterized in a fleshy fruit. That said, tomato has become a model for examining how altering TCA cycle enzymes influences fruit development and metabolite levels. Indeed, the TCA cycle has been subjected to saturated transgenic downregulation of each enzyme constituting this pathway (Nunes-Nesi et al. 2011), with downregulation of fumarase, mitochondrial malate dehydrogenase, and OGDH having particularly profound effects on fruit growth and development (Nunes-Nesi et al. 2005; Nunes-Nesi et al. 2007; Araújo et al. 2011; Centeno et al. 2011). As discussed in detail below, further study revealed that altered interconversion of pyruvate and malate in the plastid or cytosol of ripening fruit invokes diverse consequences on sugars but similar effects on cellular organic acid metabolism and transitory starch accumulation (Osorio et al. 2013).

The phenotypic results observed in the *slbtpc*-edited lines revealed a clear reduction in fruit biomass; edited fruits were significantly smaller and lighter than WT fruits and required several more days to reach red stages. This underscores the contribution of Class-2 PEPC anaplerotic flux to fruit development. Alteration of sugar content, via the downregulation of vacuolar acid invertase, resulted in increased sucrose with smaller fruits (Klann et al. 1996), a phenotype similar to what we observed in the *slbtpc* edited fruits. Altering malate levels, via the downregulation of fumarase or the mitochondrial malate dehydrogenase, did not alter the fruit size as we obtained on the *slbtpc*-edited fruits. Likewise, antisense inhibition of the OGDH complex significantly reprogrammed organic acid and amino acid profiles and accelerated ripening, yet those lines maintained WT fruit dimensions and biomass (Araújo et al. 2012b). The lower malate content in fruits of our *slbtpc* lines may impair vacuolar osmotic potential, limiting cell turgor and expansion, as malate is known to regulate fruit water relations and cell enlargement (Carrari et al. 2006). Malate acts not only as a metabolic intermediate but also as an osmolyte that drives vacuolar water influx and cell expansion during the rapid growth stage of fruit development. Adequate levels of malate support turgor-mediated cell enlargement and supplies OAA for the TCA cycle and gluconeogenesis, as part of the malate pool is known to be interconverted into hexoses (Schouten et al. 2016). Downregulation of the vacuolar proton-pumping ATPase showed that disrupted acid compartmentalization reduces cell expansion rates and final fruit size, highlighting the interplay between malate compartmentation, pH homeostasis, and growth (Amemiya et al. 2006).

In our study, malate and citrate levels were reduced, and the edited fruit exhibited delayed ripening and downregulation of ripening-associated genes, such as ethylene-responsive transcription factors, cell wall-modifying enzymes, and sugar transporters. Malate has been proposed to interact with the ethylene signaling, possibly via changes in cytosolic pH, redox state, or metabolic intermediates that modulate hormone biosynthesis and perception (Centeno et al. 2011; Batista-Silva et al. 2018). The downregulation of cell wall genes and ethylene-responsive factors in our *slbtpc*-edited lines supports this idea, linking impaired malate synthesis to suppressed ripening transcriptional program.

Together, these findings underscore that while partial modulation of TCA enzymes often triggers homeostatic compensation, complete loss of the BTPC subunit more profoundly disrupts fruit growth, revealing a unique contribution of the malate-insensitive Class 2 PEPC complex to biomass and organic acid accumulation in developing tomato fruit. During tomato ripening, there is a marked decrease in the organic acid content of the pericarp tissue (Knee and Finger 1992; Carrari et al. 2006). This decrease could be brought about either by a restriction of their synthesis or an enhanced metabolism, potentially initially catalyzed by NAD ^+^ - or NADP ^+^ -dependent ME, or PEPCK (Osorio et al. 2013). However, this is complicated by the fact that Class 1 PEPCs composed of PTPC subunits are potently inhibited by malate (O’Leary et al. 2011a; Ting et al. 2017). The metabolic shifts observed in the red-stage of the *slbtpc*-edited plants indicate a stable and long-term reprogramming of fruit metabolism consistent with *in vitro* studies demonstrating that the activity of Class-2 PEPC hetero-octameric complexes is greatly desensitized to malate inhibition (Blonde and Plaxton 2003; O’Leary et al. 2009, 2011b; Park et al. 2012; Ting et al. 2017). These metabolic changes are largely the opposite of those previously found in *pepck* RNAi lines (Osorio et al. 2013). This observation is not unsurprising given that PEPCK catalyzes the reverse reaction of PEPC, converting oxaloacetate (and ATP) into PEP (and ADP and CO_2_) in the gluconeogenic direction. The suppression of PEPCK by RNAi led to an accumulation of malate (in Breaker +5 days, not in Breaker +10 days) and a reduction in sugars such as glucose and fructose (Osorio et al. 2013; Huang et al. 2015). These results are directly in contrast to what we observed in our *slbtpc-*edited fruits, as the repression of BTPC (and hence formation of Class-2 PEPC complexes) is reducing OAA flux into the TCA cycle, ultimately reducing malate and citrate levels, whereas *pepck*-RNAi prevents malate catabolism, resulting in an increase in its levels. The reduced carbon flow into the TCA cycle in the *slbtpc*-edited lines likely results in the C being concentrated within sugars, whereas in the *pepck*-RNAi, carbon is rather “trapped’ into malate. As a further consequence, the *slbtpc* gene-edited lines display an increase in all amino acids, while the *pepck*-RNAi lines only displayed an increase in glutamate but reduced OAA-derived amino acids—namely Asp, Asn, Lys, Pro, Trp, and Met.

Whilst not directly measured here, based on previous studies, it can be hypothesized that the sustained reduction in organic acid levels and coincidental enhancement of sugar and nitrogen metabolism will influence further key physiological processes, including fruit softening, pigment accumulation, and volatile compound synthesis (Carrari et al. 2006; Fernie and Martinoia 2009; Sweetman et al. 2009; Centeno et al. 2011). The strong modulation of cell wall–related metabolism–related genes observed in the *slbtpc*-edited lines aligns with the phenotypic traits of smaller fruits with delayed softening. Although transcript-level data alone cannot confirm mechanical alterations in wall properties, the coordinated repression of expansins, polygalacturonases, and pectate lyases, combined with the induction of CesA, supports a scenario where the balance between wall loosening and synthesis is disrupted. Such transcriptional reprogramming has been associated with reduced cell expansion and firmer texture in tomato (Brummell and Harpster 2001; Uluisik et al. 2016). Downregulation of expansins and pectin-degrading enzymes has been linked to decreased wall plasticity, slower softening, and prolonged ripening (Gwanpua et al. 2014; Cosgrove 2015). It is important to note that cell-wall-related genetic manipulation does not always produce proportional effects on fruit size or firmness. For example, it was described that the double knock-out of PG and PL in tomato resulted in firmer fruits with no differences in size (Ortega-Salazar et al. 2024). Dedicated physiological analysis will be required to determine whether the transcriptional shifts we observe translate to changes in the wall rigidity, elasticity, or disassembly during ripening.

The integrative model presented in Fig. 6 illustrates how these transcriptional shifts might interact with sugar metabolism to influence ripening progression. Reduced expression of carbohydrate transporters (SWEETs) and enzymes such as myo-inositol oxygenase (MIOX), UDP-Ara 4-epimerase (UAE), and UDP-D-glucuronic acid decarboxylase (UXS) could limit the flux of sugar precursors required for wall remodeling, whereas increased CesA activity might reinforce cellulose deposition. Together, these changes could contribute to a stiffer cellular matrix, consistent with delayed softening and prolonged ripening time in *slbtpc*-edited fruits. Future work assessing cell wall composition, firmness, or polysaccharide architecture would be valuable to substantiate these transcriptomic inferences.

*In summary*, the presence of heteromeric Class-2 PEPC complexes in tomato fruit suggests an adaptive mechanism to sustain PEPC activity despite the high malate environment. Tomato fruit likely utilizes malate-insensitive Class-2 PEPC to maintain anaplerotic carbon fixation and respiratory CO₂ refixation during its development. This idea is reinforced as many malate-accumulating tissues (e.g., castor endosperm, cassava tubers, cucumber and tomato fruit) exhibit strong BTPC expression, hinting at a conserved role for BTPC in supporting the metabolism of fleshy, malate-rich sink tissues (Ting et al. 2017). The reduced malate inhibition of BTPC-containing Class-2 PEPCs would be particularly advantageous in tomato fruits, where malate levels drop only late in ripening (Carrari et al. 2006); throughout development, active PEPC could refix CO₂ while generating OAA/malate for the TCA cycle and gluconeogenic pathways, thereby buffering the fruit's carbon economy. Notably, O’Leary et al. (2011a) and Plaxton and colleagues have emphasized that PEPC's anaplerotic role is crucial for fueling fruit metabolic processes (O’Leary et al. 2011a; Ting et al. 2017), and the BTPC isoform may enhance this role under conditions that would inhibit “normal’ Class-1 PEPCs (high malate, variable phosphorylation states, etc.). As such, our study reveals an important role for BTPC in tomato fruit ripening and development, highlighting the role of its malate insensitivity within these crucial physiological processes.

## Materials and methods

### Plasmid construction

For specific mutation of *SlBTPC* via CRISPR/Cas9, the target site was selected using breaking-cas (Oliveros et al. 2016) and was designed to target the end of the first exon (Table S3, Fig. S3). The CRISPR vector was constructed as described in (Reem and Van Eck 2019). Briefly, a 20 bp oligo was cloned into the *pAGM4723* binary plasmid via Golden Gate ligation. *Agrobacterium tumefaciens* GV2260 was used for stable transformation of tomato, which was performed according to McCormick et al. 1986). Genomic DNA was extracted using a CTAB protocol to validate the mutated lines. The mutation was validated first with a restriction enzyme analysis to screen the transformants and subsequently verified by sequencing. The primers used for plasmid construction and mutation analysis are listed in Table S3.

### Plant material

Tomato (*Solanum lycopersicum* var. Moneymaker) plants were used for all the experiments. Plants were grown in a greenhouse under long-day conditions (16 h light/8 h dark photoperiod) at a controlled temperature of 22–18 °C as described in (Carrari et al. 2006). Flowers were tagged on the day of anthesis, and fruits were harvested at different timepoints post-anthesis.

### Phenotypic analysis

Ripening time was determined by tagging individual flowers on the day of anthesis and harvesting fruits on different days of growth and ripening. At least 5 different plants per line were grown. For phenotypic analysis, at least 20 fruits per line and stage were used for different measurements. Fruits were individually weighed, and the size was recorded by measuring the longitudinal and transversal size with a ruler. Fruit pericarp samples were harvested, frozen in liquid nitrogen, and stored at −80 ^o^C until further analysis. Samples for protein extraction were lyophilized prior to use.

### Protein extraction

Lyophilized tomato fruit was ground to a powder in a mortar containing a small amount of sea sand and homogenized (1:7; w/v) in 500 mM Tris-HCl (pH 7.5) containing 1 mM EDTA, 0.1% (v/v) Triton X-100, 20% (v/v) glycerol, 10 mM MgCl_2_, 5 mM thiourea, 1% (w/v) poly(vinylpolypyrrolidone), 5 mM potassium bicarbonate, 10 mM L-ascorbic acid, 1 mM phenylmethylsulphonyl fluoride, 2 mM 2,2′-dipyridyl disulfide, and 5 μl/mL ProteCEASE-100 (G-Biosciences). Homogenates were centrifuged at 4 °C and 15,000 *g* for 10 min, and supernatants were syringe-filtered through 0.45 µm PVDF membranes. Clarified extracts were immediately used for PEPC activity assays, incubated for 3 min at 100 °C in SDS-PAGE sample buffer, or frozen in liquid N_2_ and stored at −80 °C for future use.

### Electrophoresis and immunoblotting

SDS-PAGE using a Bio-Rad Protean III mini-gel apparatus and immunoblotting were performed as previously described (Blonde and Plaxton 2003). Anti-castor oil seed PTPC/RcPPC3 and BTPC/RcPPC4 immune sera (anti-PTPC and anti-BTPC, respectively) were raised in rabbits and the respective IgGs affinity purified using Pierce Protein A Chromatography Cartridges (ThermoFisher Scientific) as previously described (O’Leary et al. 2009; Ting et al. 2017). Immunoreactive polypeptides were visualized using an alkaline phosphatase-linked secondary antibody and chromogenic detection as previously described (Blonde and Plaxton 2003).

### Co-immunoprecipitation

Affinity-purified anti-PTPC-IgG (5 µg in 10 µL) was coupled to 400 µL (4 mg) of Surebeads^TM^ Protein-G Magnetic Beads (Bio-Rad), following the manufacturer's instructions. Equivalent aliquots (i.e., 205 µL) of the anti-PTPC IgG-coupled Surebeads^TM^ were then incubated for 1 h at 25 °C with clarified extracts (1 mL each) prepared from immature green WT or L8 *slbtpc*-edited fruit as described above. Beads were thoroughly washed, and bound proteins were eluted with 50 µL of 20 mM glycine-HCl (pH 2.0). The respective eluents were immediately neutralized with 5 µL of unbuffered 0.5 M Tris and subjected to SDS-PAGE. The gels were either stained with Coomassie Brilliant Blue G-250 for total protein detection or subjected to anti-PTPC and anti-BTPC immunoblotting.

### Sequencing and data analysis

Total RNA samples were extracted from WT and CRISPR/Cas9 edited lines with three biological replicates per sample, from pericarp tissue at the mature green stage, using the Machinery Nagel NucleoSpin RNA Plant following the manufacturer's procedure. RNA samples were sent to Azenta GmbH (Leipzig, Germany) for the generation of >20 M paired-end mRNA reads. Samples were subjected to the strand-specific RNA library preparation using polyA-selection and 2 × 150 bp sequencing on an Illumina NovaSeq platform.

Raw reads were pre-processed to filter out the sequencing adaptors and reads shorter than 80nt using Trimmomatic v0.39 (Bolger et al. 2014) with settings “TruSeq3-PE.fa:2:30:10:2:True HEADCROP: 36 MINLEN:80”. Transcript-level quantification of the expression was performed via mapping of the filtered libraries against *S. lycopersicum* indexed reference (ITAG4.1_cDNA, https://solgenomics.net/ftp/tomato_genome/annotation/ITAG4.0_release/) using salmon v1.10.1 with settings “-l A”, to allow automatic inference of the library type, and “–validateMappings”, for selective alignment and more sensitivity. To estimate the expression level of mRNA coding genes in wildtype and mutant lines, and following differential expression analysis, edgeR v3.36.0 (Chen et al. 2016) was employed by benefiting from functions provided by the tximport pipeline. Comparison between expression profiles, versus wildtype as reference, was conducted using glmQLFTest (Genewise Negative Binomial Generalized Linear Models with Quasi-likelihood Tests) (Chen et al. 2016; Lun et al. 2016). DEGs were selected by an FDR cutoff of 20% and a log2 fold change of at least one. DEGs were subjected to Mapman4 functional annotation using the online tool Mercator4 v6 (Schwacke et al. 2019) to obtain a detailed description of the genes. An overview of the top 10 enriched functional bins was gained using over-representation analysis for the DEs passed filtering based on the following criteria: a. either up- or down-regulated in all lines, b. absolute log FC ≥ 1 in at least one of the lines.

### Metabolomic analysis

For determination of the levels of primary metabolites, 100 mg of frozen material was ground to a fine powder using a retch mill. Samples were extracted as described in (Lisec et al. 2006). Briefly, 300 μL of methanol (with ribitol 0.2 mg/mL) was added as an internal standard to the samples, and they were subsequently incubated at 70 °C for 15 min, followed by 200 μL of chloroform and 300 μL of water. The polar fraction was dried under vacuum, and the residue was derivatized in 40 μL of 20 mg/mL methoxyamine hydrochloride in pyridine at 37 °C for 30 min, followed by 70 μL of MSTFA at 37 °C for 30 min. A Multi-Purpose autosampler (Gerstel GmbH & Co.KG) was used to inject samples into a gas chromatograph interfaced to a time-of-flight mass spectrometer (viz., a Pegasus HT TOF-MS instrument from LECO Corporation). Helium at a constant flow rate of 2 mL/s was used as a carrier gas, and gas chromatography was performed on a 30 m DB-35 column. The injection temperature was 230 °C, and those of the transfer line and ion source were both 250 °C. The initial temperature of the oven (85 °C) was raised 15 °C/min to a final temperature of 360 °C. After a solvent delay of 180 s, mass spectra over the m/z range 70–600 were recorded at a rate of 20 scans/s. All data were also processed using Xcalibur 4.0 software (Thermo Fisher Scientific, Waltham, MA, USA) to verify the metabolite identification and annotation. Identification and annotation of detected peaks followed the recommendations for reporting metabolite data (Fernie et al. 2011; Alseekh et al. 2021). Statistical analyses were performed on MetaboAnalyst 5.0 (https://www.metaboanalyst.ca/; Pang et al. 2024).

### Statistical analysis

Statistical differences among genotypes were analysed using one-way ANOVA followed by Dunnett's post-hoc test to compare each mutant line with the wild-type control. Adjusted *P*-values are reported, and significance is indicated as follows: *P < 0.05; **P < 0.01, ***P < 0.001. ****P < 0.0001. All analyses were performed using GraphPad Prism v9.0.0.

### Accession numbers

Sequence data from this article can be accessed at SRA under project number PRJEB94930.

## Supplementary Material

kiag026_Supplementary_Data

## Data Availability

Sequence data from this article can be accessed at SRA under project number PRJEB94930.
