# Supplementary material for: Malate matters: disrupting bacterial-type phosphoenolpyruvate carboxylase (BTPC) rewires tomato fruit development
Source: Plant Physiol. 2026 Jan 28;201(1):kiag026. doi: 10.1093/plphys/kiag026 (PMC13172252; doi:10.1093/plphys/kiag026)
Supplement: kiag026_Supplementary_Data [file kiag026_supplementary_data.zip › supplementarys.pdf]

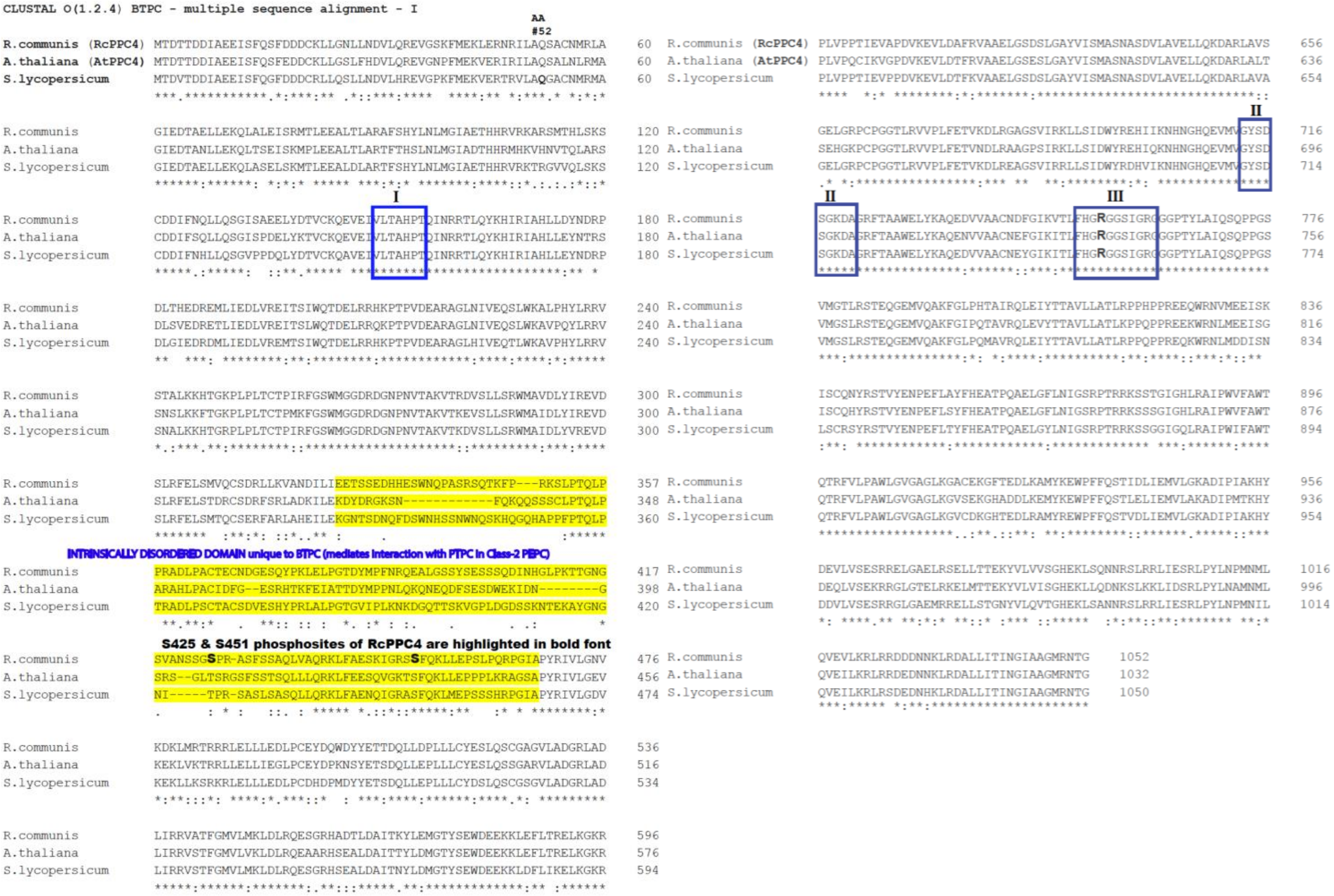

Supplementary Figure S1. Amino acid sequence alignment of SlBTPC with castor and Arabidopsis BTPC (RcPPC4 and AtPPC4, respectively) . BTPCs highly divergent approximate 10 kDa domain predicted to exist as an intrinsically disordered region is highlighted in yellow. Experimentally verified in vivo (inhibitory) phosphosites of castor BTPC are highlighted in bold.

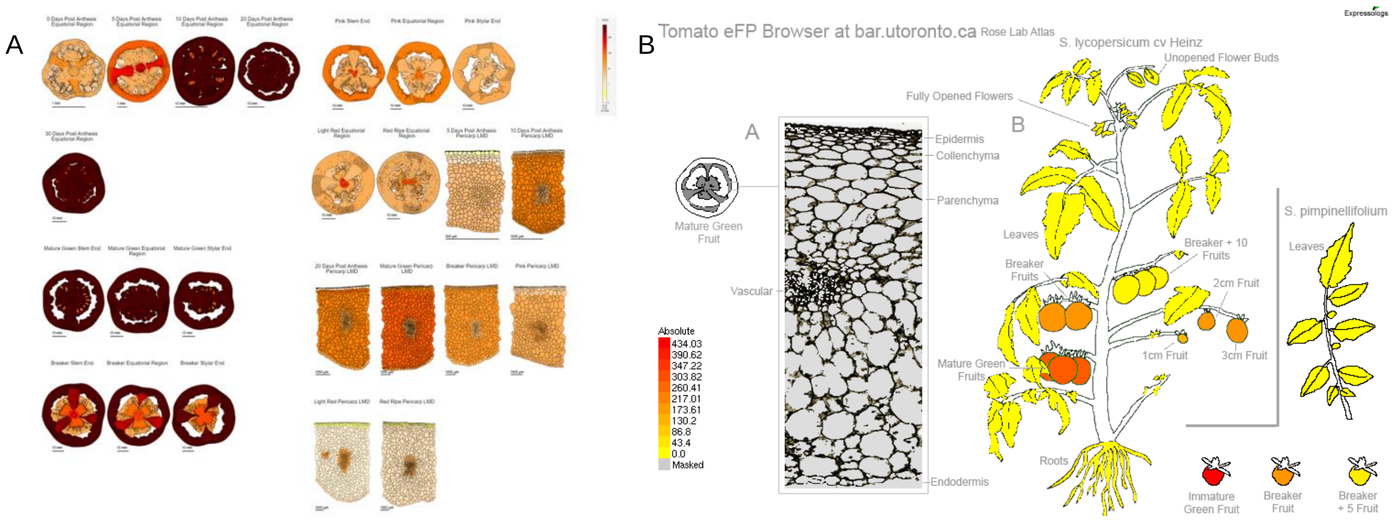

Supplementary Figure S2. Expression pattern of SlBTPC gene in open databases. SlBTPC peaks at mature green stages as reported in Ting et al., 2017. Data obtained from Shinozaki et al. 2018 (A) and Wease et al., 2013 (B).

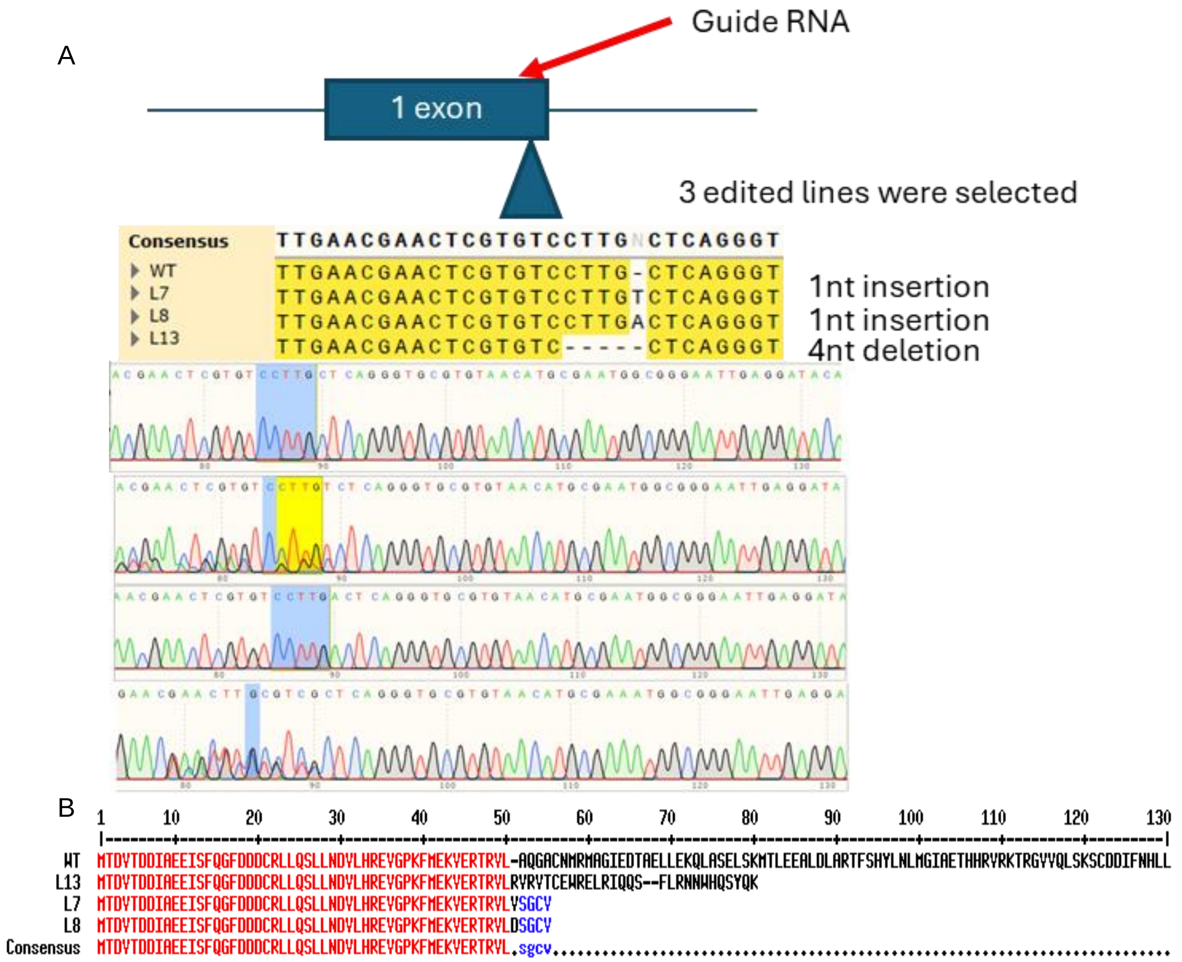

Supplementary Figure S3. Sequence analysis from *slbtpc*-edited tomato plants. A) Guide DNA was targeted to edit the end of the first exon of the gene. Zoom up shows the DNA sequence analysis from the different homozygous plant from the chromatogram. The three edited lines presented 1bp or 4bp deletion. B) Protein alignment with deducted SlBTPC sequences from WT or edited plants. Edited plants produced truncated proteins at position 54 and 80.

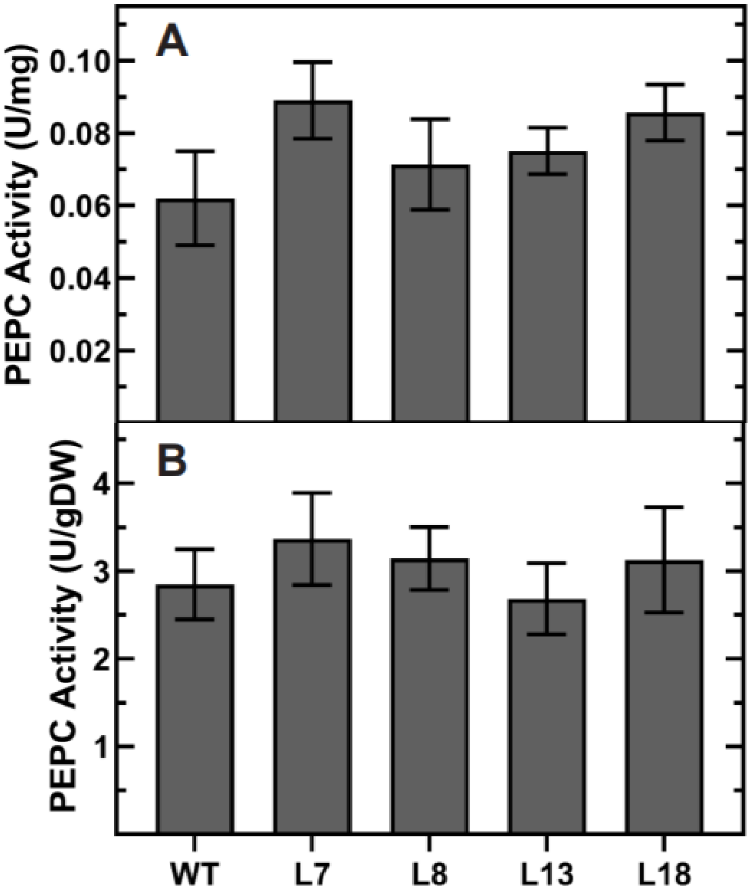

Supplementary Figure S4 . PEPC activity of clarified WT and *slbtpc*-edited fruit. Protein extracts in U/mg protein (A) and U/g dry weight (B). All values in panels A-B represent the mean ± SEM of n = 3 biological replicates. No significant differences in activity were found between WT and the various transgenic lines ($P > 0.05$; one-way ANOVA)

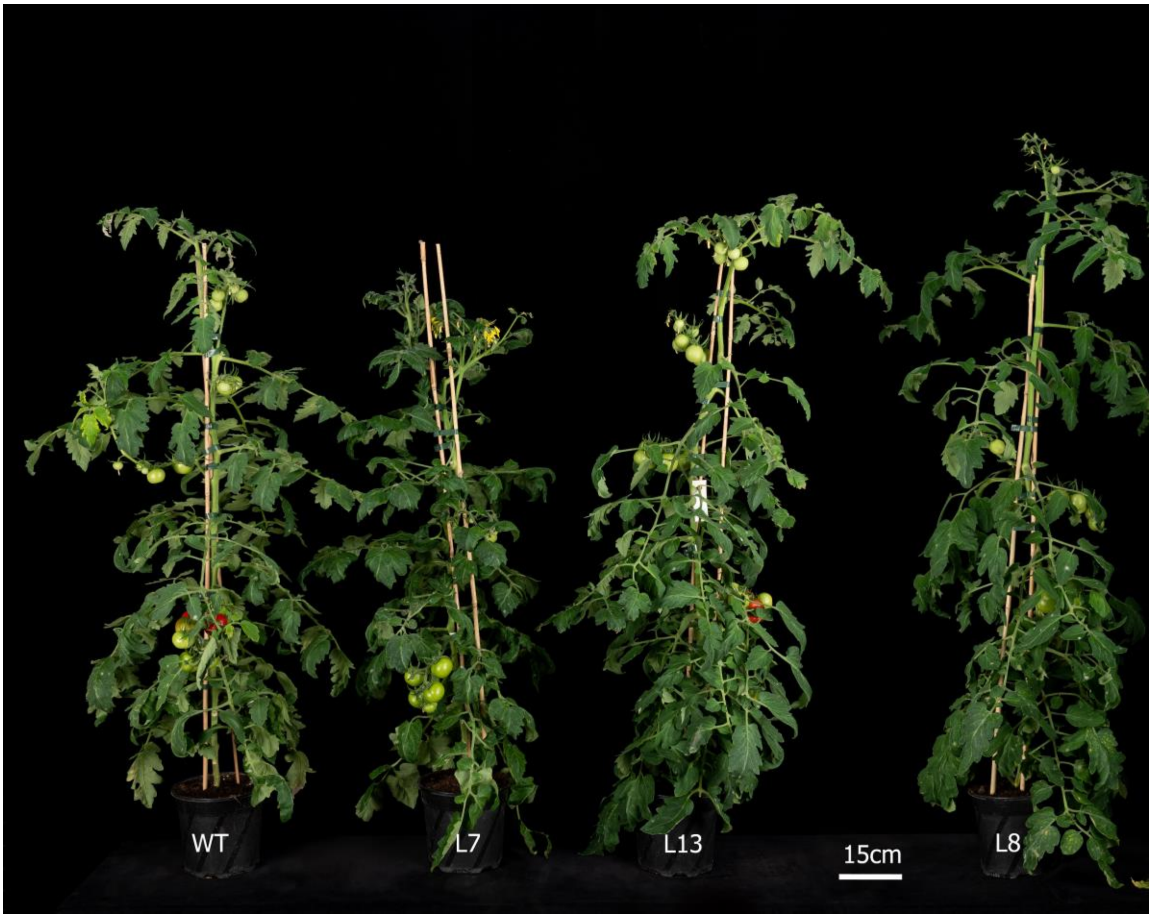

Supplementary Figure S5 . Overview of WT and *slbtpc*-edited plants. No clear phenotypic differences were observed in vegetative tissues.

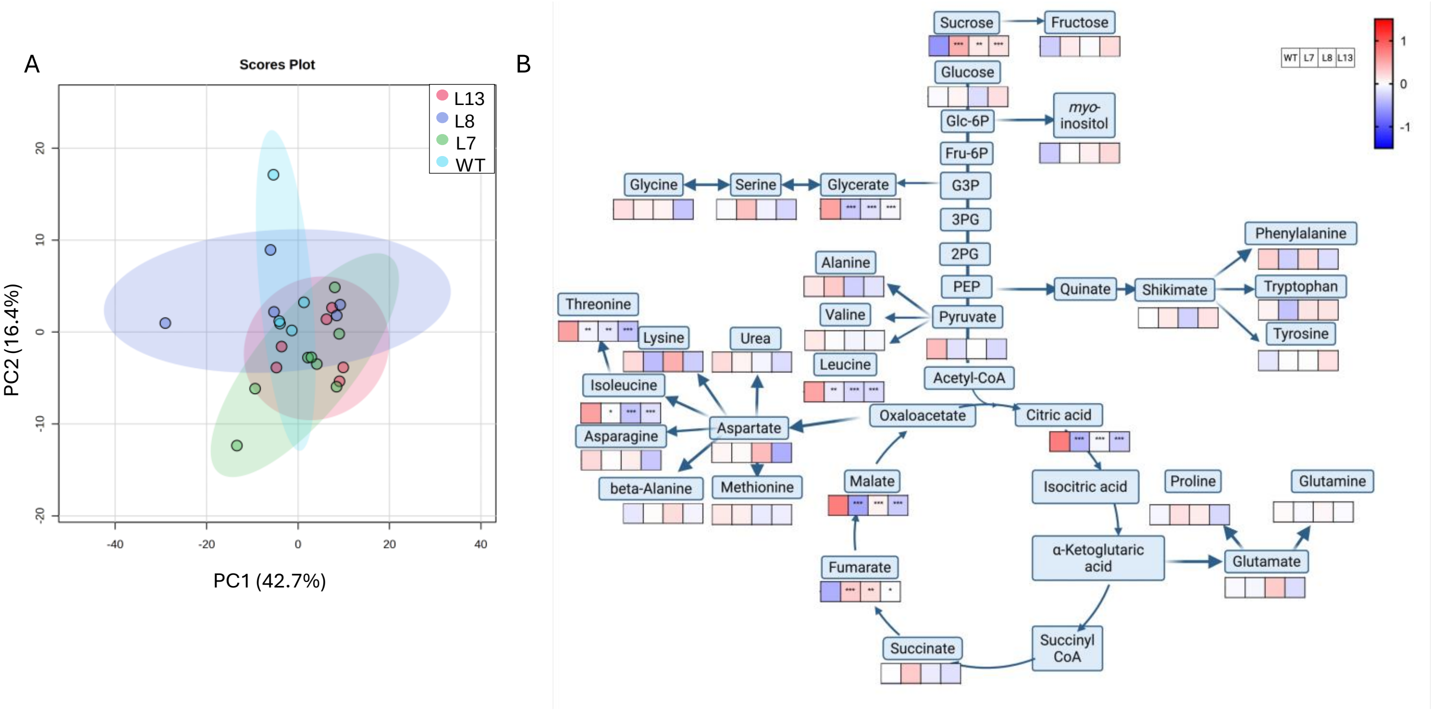

Supplementary Figure S6. Metabolism overview of immature green fruits of WT and *slbtpc* CRISPR/Cas9 lines. A) PCA analysis exhibited no clear differences between lines. B) Primary metabolites from GC-MS analysis. Pools of three fruits per plants and five plants per line were used. Metabolite levels were log transformed and pareto scaled for normalization. The scale ranges from low relative abundance (blue) to high relative abundance (red). Stars indicate statistical differences of the mean of each metabolite when compared to the WT after a t-test student * p<0.05 ** p<0.01 ***p<0.001.

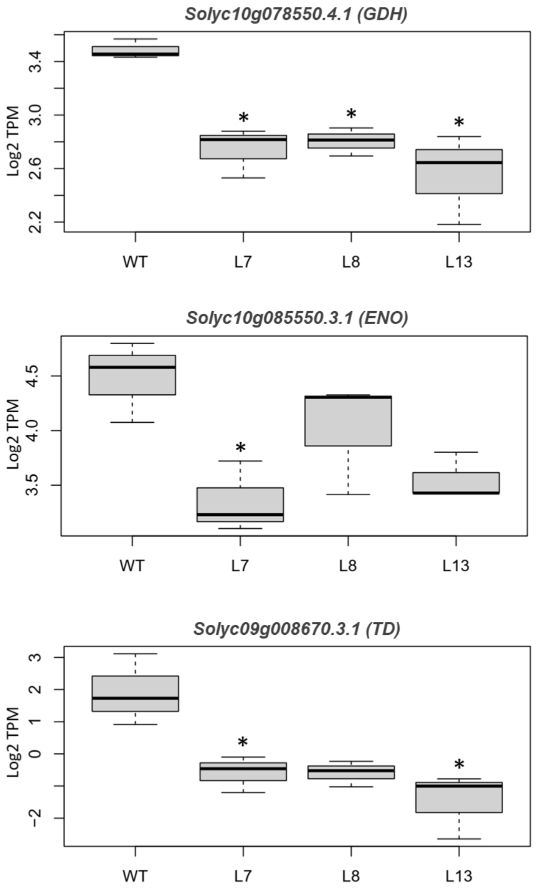

Supplementary Figure S7 . Genes encoding key enzymes in the TCA cycle and organic acid metabolism.* indicates FDR<0.05. Data are shown as Box-and-Whisker plots (line = median, box = 25–75th percentile, whiskers = min–max). Three biological replicates per line were used for RNA-Seq.

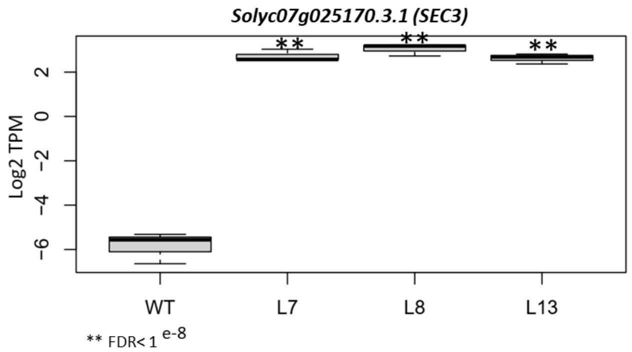

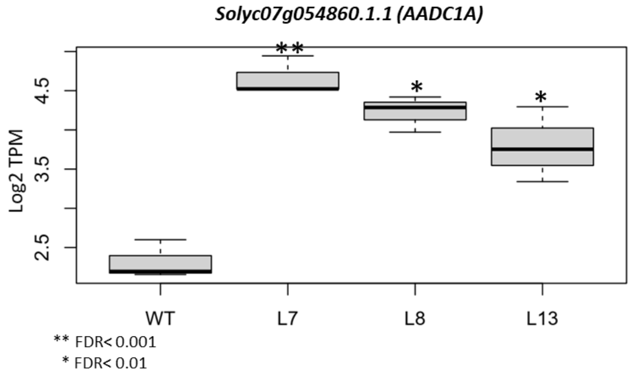

Supplementary Figure S8 . Genes encoding the strongly upregulated genes, exocyst complex components (SEC 3) and aromatic amino acid decarboxylase (AADC1A). Data are shown as Box-and-Whisker plots (line = median, box = 25–75th percentile, whiskers = min–max). Three biological replicates per line were used for RNA-Seq.
